# Prognostic value of ^99m^Tc-ECD brain perfusion SPECT in patients with atrial fibrillation and dementia

**DOI:** 10.1186/s13550-019-0589-3

**Published:** 2020-01-10

**Authors:** Hidenobu Hashimoto, Rine Nakanishi, Sunao Mizumura, Yukiko Hashimoto, Yuriko Okamura, Kyoko Yamanaka, Takanori Ikeda

**Affiliations:** 1 Department of Cardiovascular Medicine, Department of Internal Medicine, Toho University Faculty of Medicine, 6-11-1, Omorinishi, Ota-ward, Tokyo, 143-8541 Japan; 2 Department of Radiology, Toho University Faculty of Medicine, Tokyo, Japan

**Keywords:** Atrial fibrillation, Dementia, ^99m^Tc-ECD brain perfusion SPECT, Prognosis

## Abstract

**Background:**

Patients with atrial fibrillation (AF) and dementia experience reduced quality of life and increased mortality. Technetium 99m ECD brain perfusion single-photon emission computed tomography (^99m^Tc-ECD brain perfusion SPECT) is a beneficial modality for diagnosing dementia and identifying high-risk patients with mild cognitive impairment. The aim of this study was to evaluate the prognostic value of brain perfusion using ^99m^Tc-ECD SPECT in patients with AF and dementia.

**Methods:**

Of a total of 405 consecutive patients diagnosed with AF as cardiac outpatients with dementia using the Mini-Mental State Examination by neurologists or psychiatrists, we identified 170 patients (81 ± 10 years) who underwent ^99m^Tc-ECD brain perfusion SPECT. Of them, 73, 73, and 24 were diagnosed with Alzheimer’s dementia (AD), vascular dementia (VD), and non-specified dementia, respectively. A multivariable Cox model was used to assess if higher *Z*-score by ^99m^Tc-ECD brain perfusion SPECT and clinical parameters were associated with major adverse cardiovascular events (MACE) including cardiac death, myocardial infarction, hospitalization for heart failure, and stroke.

**Results:**

During a mean follow-up of 1258 ± 1044 days, 62 MACE occurred. There was no significant difference in MACE between AD and VD (33%, vs. 44%, *p* = 0.153). The multivariable Cox model confirmed that the higher *Z*-score of temporo-parieto-occipital lobe was associated with increased MACE compared to the lower group (HR 2.521, 95% CI 1.465–4.337, *p* < 0.001).

**Conclusion:**

This study demonstrated that decreased cerebral blood flow in the temporo-parieto-occipital lobe could be a potential prognostic value in patients with both AF and dementia.

## Background

Atrial fibrillation (AF) is the most common cardiac arrhythmia, and it results in reduced quality of life, functional status and cardiac performance [1]. AF is a well-recognized risk factor for cardiovascular disease as well as cerebrovascular disease [2]. It is reported that the prevalence of AF and cognitive disorders is increasing as the older population increases [3]. Patients with cognitive disorders also experience a lower quality of life and increased mortality [3, 4]. Technetium 99m ECD brain perfusion single-photon emission computed tomography (^99m^Tc-ECD brain perfusion SPECT) is a beneficial modality for diagnosing dementia and identifying high-risk patients with mild cognitive impairment [5]. The easy *Z*-score imaging system (eZIS) is one of the statistical analysis methods for automated diagnosis of brain perfusion SPECT images. It can be used to investigate the regional cerebral blood flow objectively and easily. SPECT with eZIS allows the identification of disease-specific patterns of regional cerebral blood flow in a three-dimensional brain magnetic resonance image, with the influence of age-related physiological regional cerebral blood flow changes avoided through normalization of data for each patient based on age-matched database for normal brains. However, there are few reports on the relationship between ^99m^Tc-ECD brain perfusion SPECT with eZIS and the prognosis of patients with AF and dementia. The aim of this study was to evaluate the prognostic value of brain perfusion using ^99m^Tc-ECD SPECT with eZIS in patients with AF and dementia.

## Materials and methods

### Patient population

**Fig. 1:**
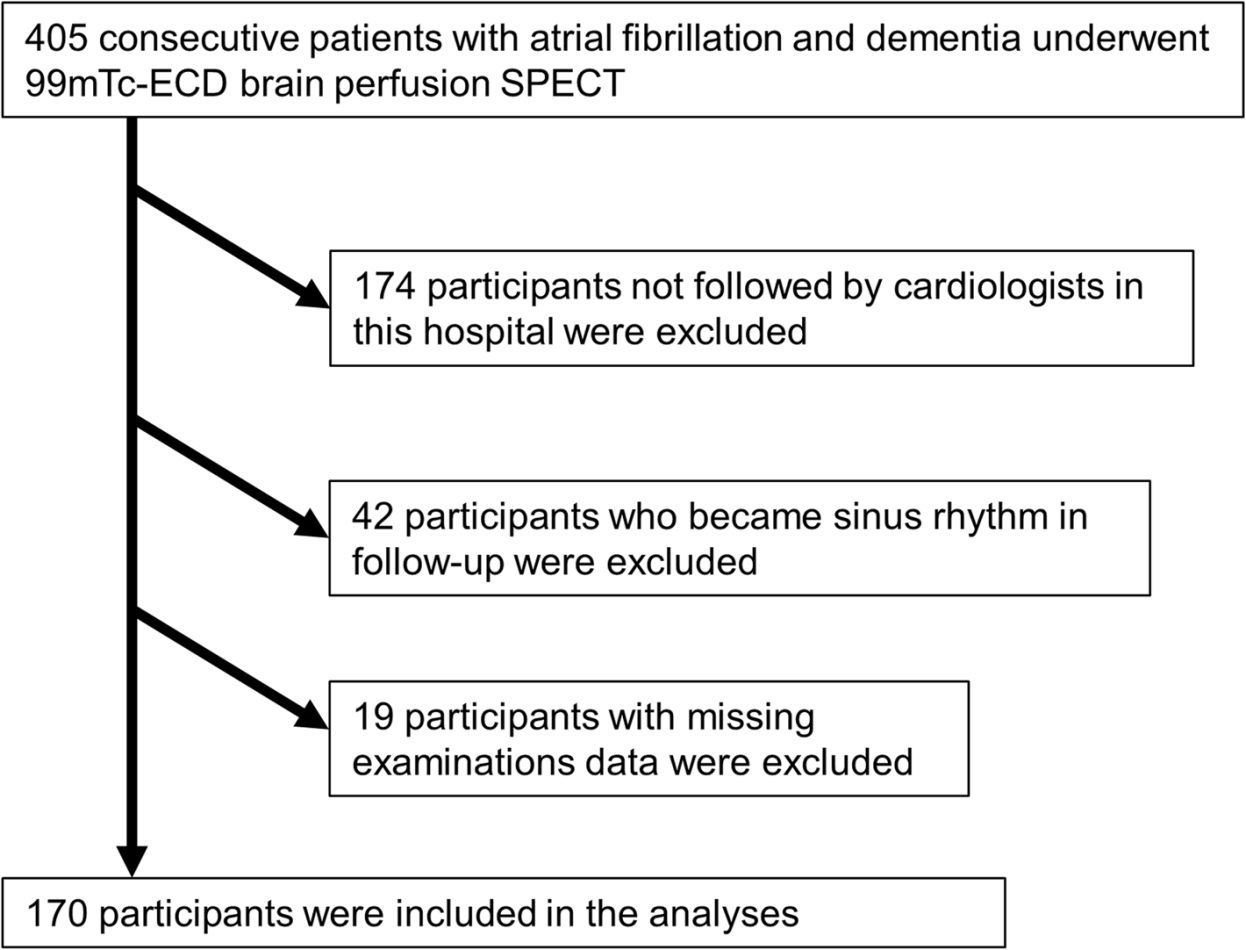
Flow chart of the patient inclusion and exclusion criteria in this study. Abbreviation: ^99m^Tc-ECD brain perfusion SPECT, technetium 99m ECD brain perfusion single-photon emission computed tomography

Between April 2003 and November 2017, 405 consecutive patients were diagnosed with AF in cardiac out-patient centers and were diagnosed with dementia by neurologists or psychiatrists using the Mini-Mental State Examination. These patients underwent ^99m^Tc-ECD brain perfusion SPECT at our institute. We identified 170 patients (81 ± 10 years) for the current study (Fig. 1). Their diagnosis was confirmed by neurologists or psychiatrists. Alzheimer’s dementia (AD) patients fulfilled the criteria of DSM-IV [6], vascular dementia (VD) patients fulfilled the criteria of ICD-10 [7], and other patients had non-specified dementia (ND). Exclusion criteria were as follows: patients who were not followed by cardiologists in our hospital, patients who become sinus rhythm during follow-up, and patients with missing examination data. We evaluated clinical characteristics including age, sex, coronary risk factors, blood biochemical data, echocardiographic data, and drug treatment. The institutional review board approved this retrospective study, and the requirement to obtain informed consent was waived (M17307).

### Assessment of AF

A 12-lead resting electrocardiogram recording (ECG-1450; Fukuda Denshi Co, Ltd., Tokyo, Japan) was performed in the study population. The electrocardiogram data were interpreted by cardiologists. This study’s inclusion criterion was an electrocardiographic documentation of AF, and the patients in this study were diagnosed with chronic AF (*n* = 117, 69%) or paroxysmal AF (*n* = 53, 31%) by cardiologists prior to undergoing ^99m^Tc-ECD brain perfusion SPECT. The CHADS2 score has been proposed and validated as a straightforward and practical stratification of stroke risk in patients with AF [8]. We retrospectively calculated the CHADS2 score for each patient by using the medical history data obtained through medical records.

### Assessment of cognitive function

A psychological and psychosocial test, the Mini-Mental State Examination was administered to the study population to evaluate cognitive function, such as attention, semantic memory, visuospatial skills, and executive function [9]. A score of 27 or under on the Mini-Mental State Examination was defined as dementia with mild cognitive impairment. The Mini-Mental State Examination was administered by neurologists and psychiatrists at our facility prior to performing the ^99m^Tc-ECD brain perfusion SPECT.

### Echocardiographic imaging

Echocardiographic images were obtained from the parasternal window for the evaluation of the left ventricular function (Vivid E9device; GE Vingmed, Horten, Norway). The left ventricular ejection fraction (LVEF) was calculated using the Teichholz formula [10].

### ^99m^Tc-ECD SPECT protocol

Before tracer administration, all subjects were laid in a supine position in a quiet room with dimmed light, with their eyes closed. Patients were injected with ^99m^Tc-ECD (600 MBq) while they were awake. Ten minutes after radiotracer injection, SPECT images were acquired using a dual-head gamma camera (Infinia, GE Healthcare, Buckinghamshire, UK) equipped with low-energy high-resolution collimators. Images were acquired with each head rotating 180° in 72 steps, at 21 s/step, and were reconstructed with a Butterworth filter (cut-off, 0.55 cycle/cm; power, 10) and displayed in a 128 × 128 matrix. Scatter correction and attenuation correction were not performed.

### Quantitation of brain SPECT images

**Fig. 2:**
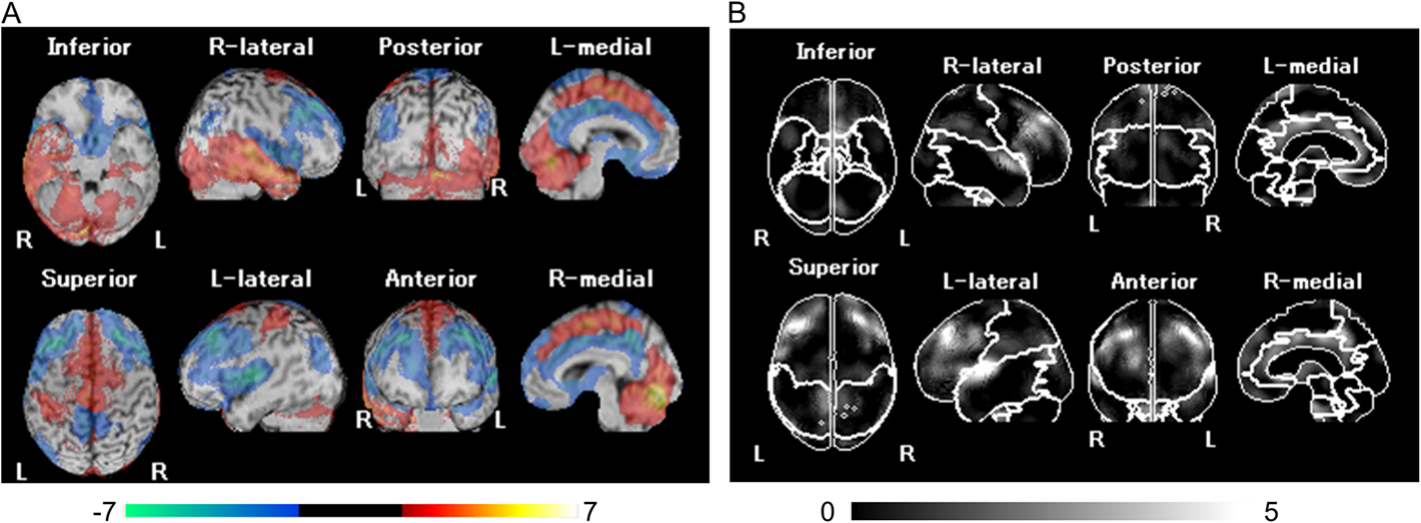
*Z*-score map by using eZIS and the segmentation map by using vbSEE. **a** Color-coding represents the scale of decrease or increase of the *Z*-score in the *Z*-score map; yellow represents the most increase area in the map. **b** Gray-scale represents the scale of decrease of the *Z*-score; white represents the most decreased area in the segmentation map. Abbreviations: eZIS, easy *Z*-score imaging system; vbSEE, voxel-based Analysis Stereotactic Extraction Estimation

SPECT images for all participants were anatomically standardized with an original ^99m^Tc-ECD template using eZIS (FUJIFILM RI Pharma Co., Ltd, Tokyo, Japan). Images from the ^99m^Tc-ECD SPECT were analyzed using a region-of-interest technique to obtain semi-quantitative parameters for tracer distribution using eZIS. A *Z*-score map for averaged SPECT image was obtained by comparison of the SPECT images with the age-matched database of normal brains. Mean and standard deviation for each voxel was obtained after anatomical standardization and voxel normalization to the global mean values using the following equation: *Z*-score = ([control mean] − [individual value])/(control SD). The *Z*-score maps were displayed by an overlay onto the topographic sections with projection, and an averaged *Z*-score was obtained from the depth of 14 mm to the surface through the rendering of an anatomically standardized MRI template (Fig. 2a). Subsequently, the severity of low flow was calculated. The *Z*-score of the frontal lobe area as an indicator of the cerebral blood flow due to arteriosclerosis, and the *Z*-score of the temporo-parieto-occipital lobe area as an indicator of cerebral blood flow related to cognitive impairment were obtained by setting the reasons automatically using a software (voxel-based Analysis Stereotactic Extraction Estimation; vbSEE) (Fig. 2b).

### Assessment of the clinical outcome

The endpoint was defined as the occurrence of major adverse cardiac events (MACE) including cardiac death (death caused by heart failure [HF], acute myocardial infarction, lethal ventricular arrhythmias, or other definitive cardiac disorders), cardiovascular events (acute myocardial infarction or unstable angina), severe HF requiring hospitalization, or stroke after undergoing the ^99m^Tc-ECD brain perfusion SPECT. Only the initial event was counted, even if patients experienced several cardiac events during follow-up. The event data were retrospectively gathered from the patients’ medical records, including in-patient and out-patient medical records.

### Statistical analysis

Data are expressed as the average ± standard deviation of the continuous variables. Continuous variables of patients with and without events were compared using the Mann-Whitney *U* test, and the categorical data were analyzed using the chi-square test. Variables including sex, age, and other factors which were significant (*p* value < 0.05) from univariable Cox regression were included in a multivariable Cox regression model to evaluate factors independently associated with future occurrence of MACE. To evaluate the clinical importance of *Z*-scores of the temporo-parieto-occipital lobe area, all patients were divided into 2 groups based on their *Z*-scores of the temporo-parieto-occipital lobe area. Each cut-off value was determined using the area under the curve (AUC) from a receiver operating characteristic (ROC) analysis based on MACE occurrences. The proportion of event-free patients was estimated using the Kaplan-Meier method and compared with the high and low *Z*-scores of the temporo-parieto-occipital lobe groups by using the log-rank test. A *p* value < 0.05 was considered statistically significant. All statistical analyses were performed using StatMate IV software version 4.01 (Advanced Technology for Medicine and Science, Tokyo, Japan).

## Results

Patient characteristics including coronary risk factors, AF parameters such as the CHADS2 score, dementia type, Mini-Mental State Examination (MMSE) score, brain natriuretic peptide (BNP), LVEF calculated using echocardiography, medication data, and *Z*-score calculated using brain SPECT are presented in Table 1. The mean age of the 170 patients was 81 ± 10 years, and 87 (51%) of them were men. Of these 170 patients, 73, 73, and 24 were diagnosed with AD, VD, and ND, respectively.

**Table 1:** Characteristics of all patients with or without MACE

	Total (*n* = 170, %)	MACE (*n* = 62, %)	No MACE (*n* = 108, %)	*P* value
Age (years)	81 ± 9	82 ± 10	81 ± 10	0.269
Male	87 (51)	31 (50)	56 (52)	0.942
Obesity (BMI ≥ 25kg/m^2^)	41 (24)	11 (18)	30 (28)	0.192
BMI (kg/m^2^)	22.3 ± 3.9	21.4 ± 3.8	22.9 ± 3.9	0.017
Diabetes mellitus	50 (29)	22 (35)	28 (26)	0.221
Hypertension	101 (59)	40 (65)	61 (56)	0.304
Dyslipidemia	40 (24)	18 (29)	22 (20)	0.200
Current smoking	67 (39)	30 (48)	37 (34)	0.070
CKD (eGFR < 60mL/min/1.73m^2^)	86 (51)	39 (63)	47 (44)	0.015
Atrial fibrillation				
Duration of AF (days)	1339 ± 1400	1262 ± 1368	1384 ± 1423	0.505
Paroxysmal AF	53 (31)	21 (34)	32 (30)	0.606
CHADS2 score	2.0 ± 1.0	2.5 ± 1.0	1.7 ± 0.9	< 0.001
Dementia				
AD/VD/ND	73/73/24	24/32/6	49/41/18	0.153
MMSE score	22.7 ± 4.0	21.7 ± 4.1	23.2 ± 3.9	0.017
Echocardiography				
LVEF (%)	64.3 ± 12.1	63.0 ± 13.7	65.1 ± 11.1	0.378
LAD (cm)	4.0 ± 0.8	4.1 ± 0.8	4.0 ± 0.8	0.217
Blood test				
D-dimer (μg/mL)	2.1 ± 1.9	2.6 ± 2.3	1.8 ± 1.5	0.004
BNP (pg/mL)	203.4 ± 332.7	326.7 ± 491.0	132.6 ± 153.4	0.002
Medications				
β-blockers	88 (52)	35 (56)	53 (49)	0.354
Calcium blockers	40 (24)	11 (18)	29 (27)	0.178
ACEI, ARB	88 (52)	38 (61)	50 (46)	0.060
Antiplatelet drugs	53 (31)	25 (40)	28 (26)	0.051
Anticoagulation drugs	122 (72)	44 (71)	78 (72)	0.861
Statins	47 (28)	21 (34)	26 (24)	0.169
Brain SPECT				
*Z*-score of Frontal lobe	2.05 ± 0.68	2.03 ± 0.72	2.05 ± 0.67	0.668
TPO-Z-score	1.41 ± 0.54	1.59 ± 0.52	1.31 ± 0.53	< 0.001

*MACE* major adverse cardiac events, *BMI* body mass index, *CKD* chronic kidney disease, *eGFR* estimated glomerular filtration rate, *AF* atrial fibrillation, *AD* Alzheimer’s dementia, *VD* vascular dementia, *VD* non-specified dementia, *MMSE* Mini-Mental State Examination, *LVEF* left ventricular ejection fraction, *LAD* left atrium dimension, *BNP* brain natriuretic peptide, *ACEI* angiotensin converting enzyme inhibitor, *ARB* angiotensin II receptor blocker, *SPECT* single-photon emission computed tomography, *TPO-Z-score Z*-score of the temporo-parieto-occipital lobe

**Fig. 3:**
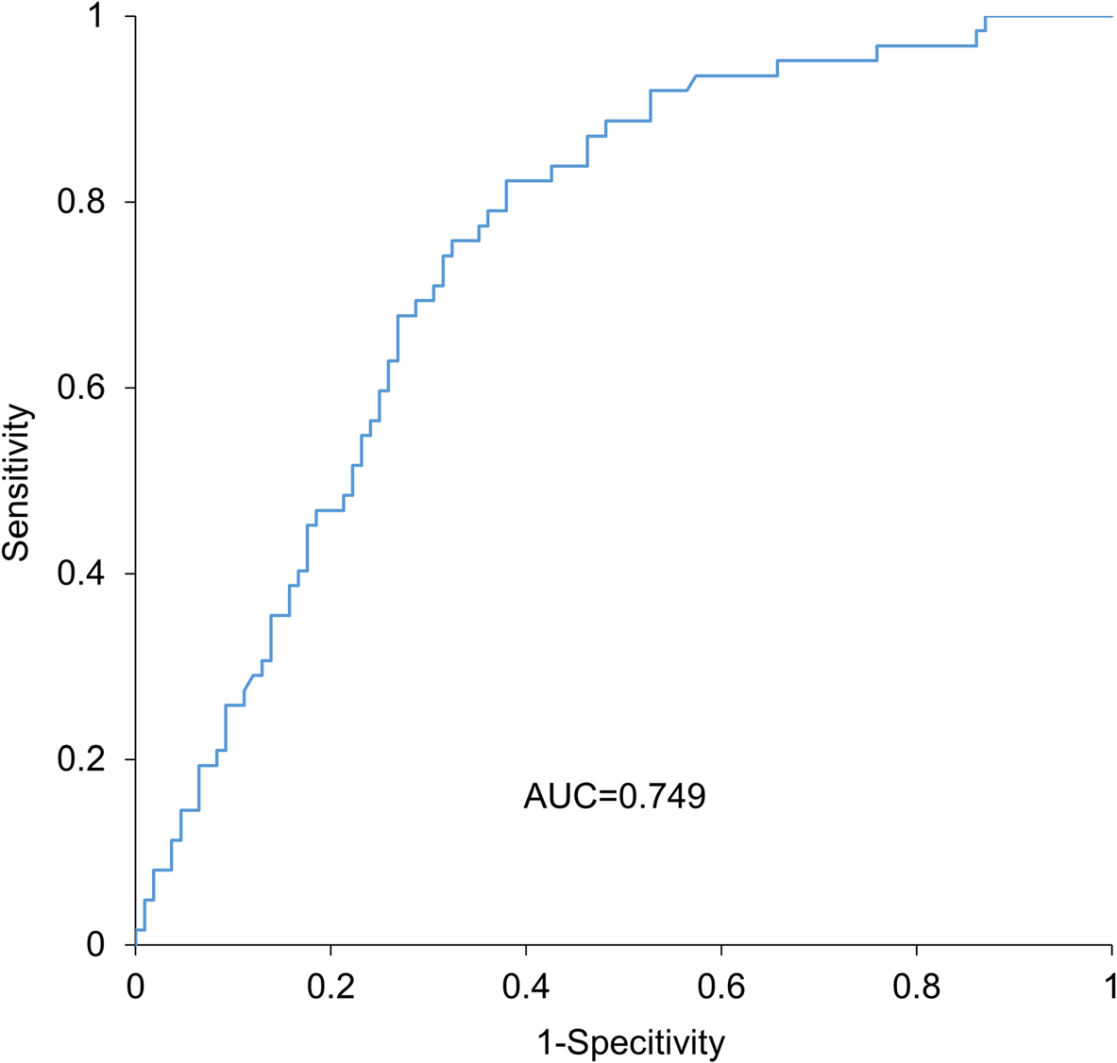
ROC analysis used for determining the cut-off score based on the occurrence of MACE. Cut-off for the high score was 1.44, and the AUC was 0.749. Abbreviations: AUC, area under the curve; ROC, receiver operating characteristic

**Fig. 4:**
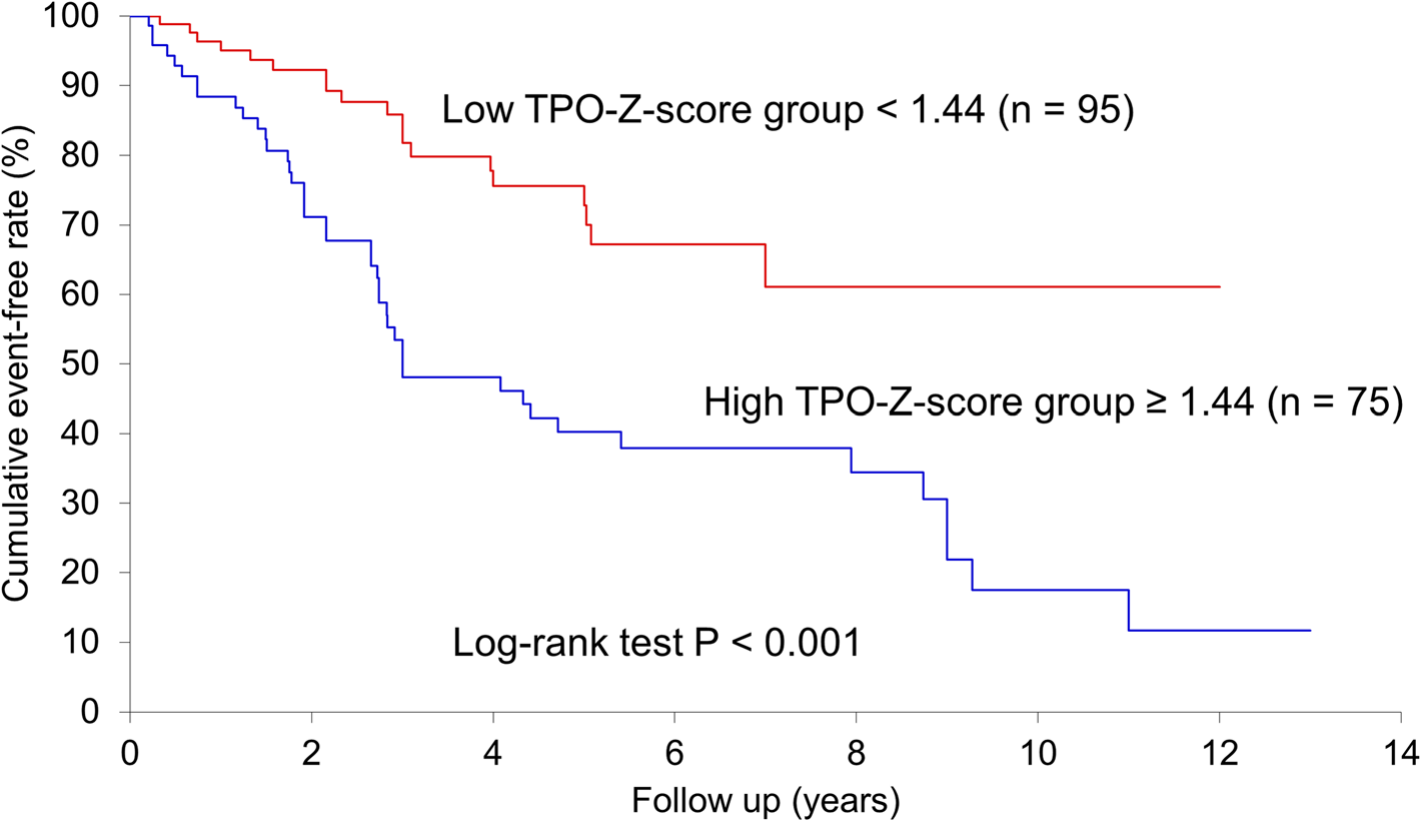
Kaplan-Meier curve in reference to MACE stratified by the *Z*-score of the temporo-parieto-occipital lobe. The *y*-axis represents the cumulative event-free rate; the rate in the lower *Z*-score of the temporo-parieto-occipital lobe group was significantly higher than that in the higher *Z*-score group (log-rank test, *P* < 0.001). Abbreviations: TPO-Z-score, *Z*-score of the temporo-parieto-occipital lobe; MACE, major adverse cardiovascular events

Overall, 62 patients (36%) experienced MACE events during 3.4 ± 2.9 years of median follow-up. Cardiac deaths occurred in 13 patients (acute myocardial infarction in 4, deterioration of HF in 9), non-fatal acute myocardial infarction in 1, severe HF requiring hospitalization in 33, and stroke in 15. Table 1 shows that chronic kidney disease (CKD), CHADS2 scores, MMSE scores, D-dimer, BNP, and *Z*-scores of the temporo-parieto-occipital lobe area were significantly higher, and body mass index was significantly lower in patients with MACE. In univariate analysis, CKD, CHADS2 scores, MMSE scores, BNP, and *Z*-scores of the temporo-parieto-occipital lobe area were found to be significant factors for MACE (Table 2). In multivariable analysis, CHADS2 scores, MMSE scores, BNP, and *Z*-scores of the temporo-parieto-occipital lobe area were significantly associated with increased MACE. The *Z*-score of the temporo-parieto-occipital lobe area was the most significant prognostic factor of MACE (Table 2). We thus investigated the clinical importance of the *Z*-score of the temporo-parieto-occipital lobe area by dividing all patients into 2 groups based on their *Z*-score of the temporo-parieto-occipital lobe. From the ROC analysis, 75 patients were assigned to the higher *Z*-score of temporo-parieto-occipital lobe group, and the remaining 95 were assigned to the lower *Z*-score group. The AUC of the ROC in predicting MACE was 0.749 for the *Z*-score of the temporo-parieto-occipital lobe. The cut-off value for higher *Z*-scores of the temporo-parieto-occipital lobe was 1.44 (Fig. 3). *Z*-scores of the temporo-parieto-occipital lobe > 1.44 had 69.4% sensitivity, 70.4% specificity, 57.3% positive predictive value, and 80.0% negative predictive value to predict MACE. Of the 62 incidents of events, 43 cases occurred in the higher *Z*-score of the temporo-parieto-occipital lobe group. The Kaplan-Meier curve demonstrated that the proportion of patients who experienced MACE was significantly higher in the higher *Z*-score of the temporo-parieto-occipital lobe group than in the lower *Z*-score group (52% [43/75] vs 21% [19/95], *p* < 0.001) (Fig. 4).

**Table 2:** Univariate and multivariable analysis for the occurrence of MACE

	Univariate analysis		Multivariable analysis	
	HR (CI)	*P* value	HR (CI)	*P* value
Age (> 75 years)	1.233 (0.644–2.362)	0.527		
Male	0.806 (0.488–1.331)	0.398		
BMI (> 25 kg/m^2^)	0.621 (0.328–1.176)	0.144		
CKD (eGFR < 60 mL/min/1.73 m^2^)	1.756 (1.045–2.949)	0.033	1.207 (0.702–2.077)	0.496
CHADS2 score (≥ 3)	2.688 (1.402–5.153)	0.003	2.275 (1.166–4.440)	0.016
MMSE score (< 23)	2.329 (1.362–3.980)	0.002	1.873 (1.090–3.216)	0.023
D-dimer (> 1.5 μg/mL)	0.907 (4.90–1.680)	0.757		
BNP (> 100 pg/mL)	2.580 (1.531–4.348)	< 0.001	2.023 (1.167–3.508)	0.012
TPO-Z-score (≥ 1.44)	2.426 (1.422–4.138)	0.001	2.521 (1.465–4.337)	< 0.001

*MACE* major adverse cardiac events, *HR* hazard ratio, *CI* confidence interval, *BMI* body mass index, *CKD* chronic kidney disease, *eGFR* estimated glomerular filtration rate, *MMSE* Mini-Mental State Examination, *BNP* brain natriuretic peptide, *TPO-Z-score Z*-score of the temporo-parieto-occipital lobe

### Case presentations

**Fig. 5:**
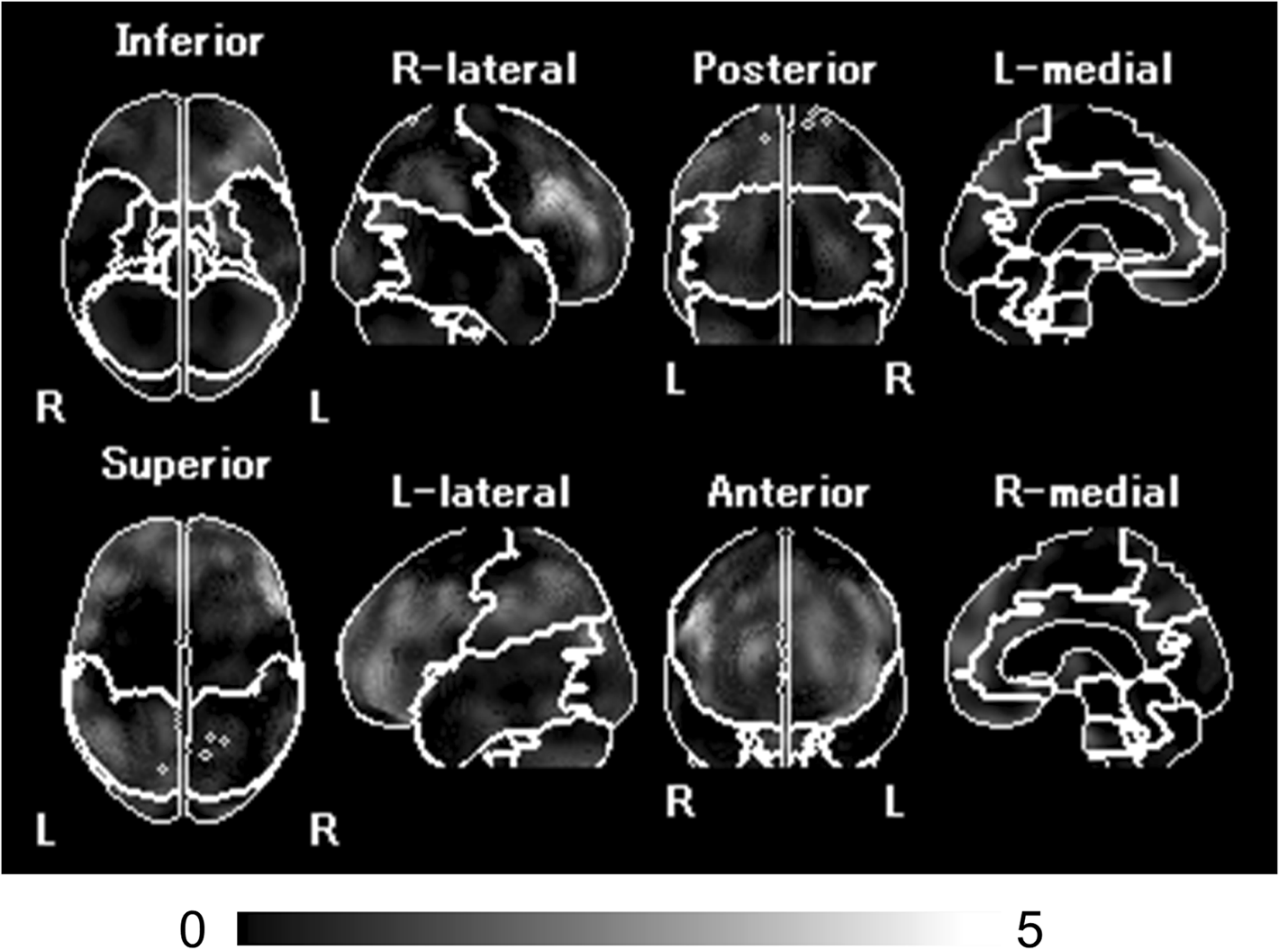
The *Z*-score map of a patient in the lower *Z*-score of the temporo-parieto-occipital lobe group. This patient had AF and AD. The MMSE score was 23, and the CHADS2 score was 2. The *Z*-score of the temporo-parieto-occipital lobe was 0.89, and the patient remained event-free for 6 years and 6 months. Abbreviations: AF, atrial fibrillation; AD, Alzheimer’s dementia; MMSE, Mini-Mental State Examination

Figure 5 shows the *Z*-score map with segmentation by using vbSEE of a typical patient with a lower *Z*-score of the temporo-parieto-occipital lobe group. The patient was a 70-year-old woman who presented with a memory disorder and had AF (CHADS2 score, 2) and Alzheimer’s dementia (MMSE score, 23). She had a history of hypertension and diabetes mellitus. She underwent ^99m^Tc-ECD SPECT to evaluate her dementia. The image showed a significant decrease in the bilateral prefrontal cortex and the bilateral parietal association areas. Her *Z*-score of the temporo-parieto-occipital lobe was 0.89. In this case, the patient remained event-free for 6 years and 6 months during the follow-up period.

**Fig. 6:**
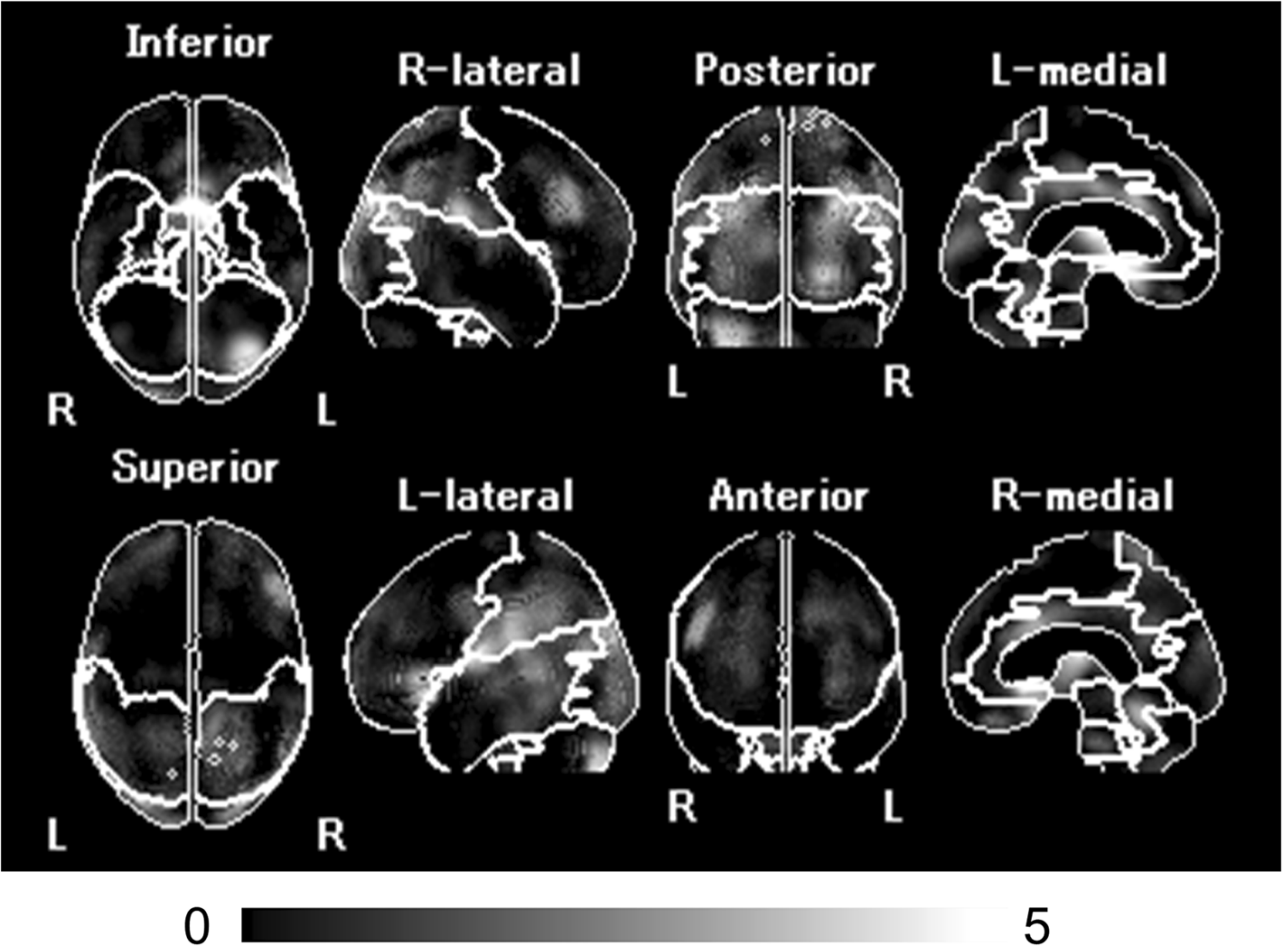
*Z*-score map of a patient in the higher *Z*-score of the temporo-parieto-occipital lobe group. This patient had AF and dementia. The MMSE score was 21, and the CHADS2 score was 4. The *Z*-score of the temporo-parieto-occipital lobe was 2.14, and the patient had cardiac death due to deterioration of HF 550 days after examination. Abbreviations: AF, atrial fibrillation; MMSE, Mini-Mental State Examination; vbSEE, voxel-based Analysis Stereotactic Estimation

Figure 6 shows the *Z*-score map with segmentation by using vbSEE of a typical patient with a higher *Z*-score of the temporo-parieto-occipital lobe group. The patient was a 60-year-old woman with AF (CHADS2 score, 4) and dementia (MMSE score, 21). She had a history of hypertension, diabetes mellitus, angina pectoris, and CKD. She underwent ^99m^Tc-ECD SPECT to evaluate the dementia. The image showed a significant decrease in the bilateral temporal parietal cortex. Her *Z*-score of the temporo-parieto-occipital lobe was 2.41. In this case, the patient had cardiac death due to deterioration of HF 1 year and 6 months after ^99m^Tc-ECD SPECT.

## Discussion

In the present study of patients with AF and dementia, we demonstrated the prognostic value of ^99m^Tc-ECD brain perfusion SPECT, which is generally used for risk assessment in patients with dementia. Our findings demonstrated that the higher *Z*-score of the temporo-parieto-occipital lobe were associated with an increase in MACE events.

### Patients with AF and dementia

The number of AF patients in the USA is about 2.3 million, and the numbers are increasing. It is predicted that more than half of patients over 80 years old will have AF in the USA [11]. It was reported that the prevalence of cognitive disorder increases with age, increasing 5% of patients in their 70s to nearly 40% of that in 90s [12]. By 2040 over 80 million people are expected to have cognitive disorder worldwide [13, 14]. Furthermore, recent data has emerged demonstrating an association between AF and dementia progression [15]. Therefore, given the high-risk nature of AF and its relationship to dementia, prediction of future cardiac risk by using a non-invasive imaging method is essential. Several prior investigations have highlighted the mechanisms responsible for the association between AF and dementia [16–19]. Cerebral microbleeds increase with age, and anticoagulation and microbleeds are associated with dementia [20, 21]. Cerebral hypoperfusion during AF may contribute to dementia. Decreased diastolic cerebral perfusion has also been associated with AF, and the irregular ventricular contraction during AF affects preload and cardiac output, which may result in a decreased mean cerebral flow [22, 23]. Therefore, evaluation of cerebral flow in AF patients by using ^99m^Tc-ECD brain perfusion SPECT may serve not only for the diagnosis of dementia types and their severity but also as a measurement of the body’s circulation due to AF.

### Prognostic value of ^99m^Tc-ECD brain perfusion SPECT in patients with AF and dementia

In the current study, the *Z*-score of the temporo-parieto-occipital lobe area showed a significant association with cardiac events based on multivariable analysis. It has been reported that the temporo-parieto-occipital lobe brain areas control cognitive function [24]. Therefore, the low accumulation of ^99m^Tc-ECD in these areas by using ^99m^Tc-ECD brain SPECT can evaluate the severity of dementia [24]. The current study suggested that the accumulation of ^99m^Tc-ECD in the temporo-parieto-occipital lobe areas may be a prognostic indicator for cardiac MACE. However, there was no significant difference in the accumulation of ^99m^Tc-ECD in the frontal lobe area between patients with and without MACE. Therefore, the low accumulation of ^99m^Tc-ECD in the frontal lobe area may indicate average brain perfusion due to AF. Patients with AF underlying coronary vascular/microvascular dysfunction have cerebral perfusion dysfunction [25]. These patients are more likely to develop HF, both systolic and diastolic, which serves as a distinct mechanism underlying reduced cerebral perfusion [26]. It is speculated that decreased perfusion of the temporo-parieto-occipital lobe area produced by AF results in a poor prognosis. In addition to the specific risks of AF, as dementia worsens, medication compliance, mental state, and activity worsen. This may lead to a poor prognosis in cardiovascular disease.

### Study limitations

This study has several limitations. The number of patients was relatively small. However, our results clearly demonstrated that higher *Z*-scores of the temporo-parieto-occipital lobe area were significantly associated with MACE events. Although cerebral infarction or brain atrophy could influence the *Z*-score, we have not performed brain MRI for all the patients in the current study. While the *Z*-score could be overestimated in VD patients, we observed that the *Z*-scores of the temporal-occipital-parietal lobe in AD patients were higher than those of VD patients (*p* = 0.0132). A further limitation was that this study retrospectively analyzed the ^99m^Tc-ECD brain perfusion SPECT data and outcomes from patients with AF and dementia. Therefore, the outcome review of medical records may have been incomplete. The prognostic impact on each etiology of dementia was not evaluated adequately due to the small sample size. Future prospective studies of large populations are required to confirm the prognostic value of *Z*-scores of the temporo-parieto-occipital lobe area in patients with AF and dementia.

## Conclusions

This study demonstrated that the decreased cerebral blood flow in the temporo-parieto-occipital lobe measured by ^99m^Tc-ECD brain perfusion SPECT could be a potential prognostic value in patients with both AF and dementia.

## Data Availability

The datasets used and analyzed during the current study are available from the corresponding author on reasonable request.

## Abbreviations

ACEI: Angiotensin converting enzyme inhibitor
AD: Alzheimer’s dementia
ARB: Angiotensin II receptor blocker
AF: Atrial fibrillation
AUC: The area under the curve
BMI: Body mass index
BNP: Brain natriuretic peptide
CI: Confidence interval
CKD: Chronic kidney disease
eGFR: Estimated glomerular filtration rate
eZIS: The easy *Z*-score imaging system
HF: Heart failure
HR: Hazard ratio
LAD: Left atrium dimension
LVEF: Left ventricular ejection fraction
MACE: Major adverse cardiac events
MMSE: Mini-Mental State Examination
ND: Non-specified dementia
ROC: Receiver operating characteristic
^99m^Tc-ECD brain perfusion SPECT: Technetium 99m ECD brain perfusion single-photon emission computed tomography
TPO-Z-score: *Z*-score of temporo-parieto-occipital lobe
VD: Vascular dementia
